# Three-dimensional thoracic aorta principal strain analysis from routine ECG-gated computerized tomography: feasibility in patients undergoing transcatheter aortic valve replacement

**DOI:** 10.1186/s12872-018-0818-0

**Published:** 2018-05-02

**Authors:** Alessandro Satriano, Zachary Guenther, James A. White, Naeem Merchant, Elena S. Di Martino, Faisal Al-Qoofi, Carmen P. Lydell, Nowell M. Fine

**Affiliations:** 1 Stephenson Cardiac Imaging Centre, University of Calgary, Calgary, Alberta Canada; 2 Division of Cardiology, Department of Cardiac Sciences, Libin Cardiovascular Institute of Alberta, University of Calgary, South Health Campus, 4448 Front Street SE, Calgary, Alberta T3M 1M4 Canada; 3 Department of Diagnostic Imaging, Cummings School of Medicine, University of Calgary, Calgary, Alberta Canada; 4 Department of Civil Engineering and Centre for Bioengineering Research and Education, University of Calgary, Calgary, Alberta Canada

**Keywords:** Computerized tomography, Strain, 3-dimensional, Aortic valve stenosis, Transcatheter aortic valve replacement

## Abstract

**Background:**

Functional impairment of the aorta is a recognized complication of aortic and aortic valve disease. Aortic strain measurement provides effective quantification of mechanical aortic function, and 3-dimenional (3D) approaches may be desirable for serial evaluation. Computerized tomographic angiography (CTA) is routinely performed for various clinical indications, and offers the unique potential to study 3D aortic deformation. We sought to investigate the feasibility of performing 3D aortic strain analysis in a candidate population of patients undergoing transcatheter aortic valve replacement (TAVR).

**Methods:**

Twenty-one patients with severe aortic valve stenosis (AS) referred for TAVR underwent ECG-gated CTA and echocardiography. CTA images were analyzed using a 3D feature-tracking based technique to construct a dynamic aortic mesh model to perform peak principal strain amplitude (PPSA) analysis. Segmental strain values were correlated against clinical, hemodynamic and echocardiographic variables. Reproducibility analysis was performed.

**Results:**

The mean patient age was 81±6 years. Mean left ventricular ejection fraction was 52±14%, aortic valve area (AVA) 0.6±0.3 cm^2^ and mean AS pressure gradient (MG) 44±11 mmHg. CTA-based 3D PPSA analysis was feasible in all subjects. Mean PPSA values for the global thoracic aorta, ascending aorta, aortic arch and descending aorta segments were 6.5±3.0, 10.2±6.0, 6.1±2.9 and 3.3±1.7%, respectively. 3D PSSA values demonstrated significantly more impairment with measures of worsening AS severity, including AVA and MG for the global thoracic aorta and ascending segment (p<0.001 for all). 3D PSSA was independently associated with AVA by multivariable modelling. Coefficients of variation for intra- and inter-observer variability were 5.8 and 7.2%, respectively.

**Conclusions:**

Three-dimensional aortic PPSA analysis is clinically feasible from routine ECG-gated CTA. Appropriate reductions in PSSA were identified with increasing AS hemodynamic severity. Expanded study of 3D aortic PSSA for patients with various forms of aortic disease is warranted.

**Electronic supplementary material:**

The online version of this article (10.1186/s12872-018-0818-0) contains supplementary material, which is available to authorized users.

## Background

Alterations in aortic biomechanical properties are recognized to occur with advancing age and in patients with primary and secondary forms of aortopathy [1, 2]. The resultant impairment in aortic function is an important contributor to global cardiovascular performance [3–5], and has been shown to have prognostic implications in specific patient cohorts, such as those with calcific degenerative aortic valve stenosis (AS) [6–8]. Indeed, associations between AS and increased aortic stiffness have been reliably demonstrated, leading to a theory that aortic disease may contribute to both symptom burden and clinical outcomes in this population [6, 8, 9]. However, optimal methods for assessing thoracic aorta biomechanics remains uncertain.

ECG-gated, contrast-enhanced computerized tomographic angiography (CTA) offers unique advantages for the study of aortic deformation. With complete 3-dimensional (3D) visualization at high isotropic spatial resolution, small displacements of the aortic wall can be resolved throughout the complex architecture. Multi-phase reconstruction of such imaging has previously been used to derive 2-dimensional (2D) strain, [10–12]. A methodology exists to perform in-vivo 3D aortic strain analysis requiring a single segmentation at end-diastole, optically and automatically tracked in 3 dimensions throughout the cardiac cycle relying on a velocity-field reconstructed for the whole dataset by means image-based feature tracking, described by Satriano et al. [13]. Another approach exists leveraging on segmentations performed throughout the cardiac cycle, described by Pasta et al. [14]. In this approach, the point cloud obtained for the end-diastolic aortic luminal surface is projected normally onto the surfaces reconstructed for other cardiac phases [14]. However, the application of the single-segmentation approach [13] specifically to multi-phase CTA is previously undescribed. The latter is of interest given an emerging desire to serially and reproducibly evaluate regional patterns of disease across specific clinical populations [15]. Furthermore, the 3D optical tracking throughout the cardiac cycle [13] allows reconstruction of the full displacement field of the mesh, beyond the displacement of each node perpendicularly to the mesh. This allows for a comprehensive quantification of deformation [13]. Facilitating such an ability to evaluate 3D deformation, Principal strain analysis has been identified as an ideal deformation measure as it endeavors to measure tissue expansion without requirement for a pre-established axis of reference, describing the dominant (or principal) direction which strain is occurring within the tissue. This establishes a geometry-independent marker that may better represent vessel wall fiber orientation [16].

Exploiting the combined advantages of ECG-gated CTA and principal strain analysis, this study aimed to assess both the clinical feasibility and reproducibility of performing 3D principal aortic strain analysis in a clinical referral population. Among patients referred for transcatheter aortic valve replacement (TAVR), a population recognized for their burden of aortic disease and routinely referred for ECG-gated CTA, we identify the capacity of this technique to visualize and quantify global values and regional patterns of aortic deformation. Internal validation of aortic strain measures against expected influences of AS severity was also explored.

## Methods

### Study population

Twenty-one consecutive patients with severe, symptomatic AS referred for TAVR were included [17]. All patients underwent pre-operative assessment with ECG-gated CTA and transthoracic echocardiography prior to TAVR. Patients with atrial fibrillation, contraindications to receiving iodinated contrast dye, and those having prior aortic or cardiac surgery were excluded. Clinical and demographic data and systemic blood pressure (measured by brachial artery cuff sphygmomanometer) were obtained at the time of echocardiography. This study was reviewed and approved by the University of Calgary Research Ethics Board, and a waiver of consent was granted for access to patient health information due to the retrospective nature of the analysis.

### Computed tomography imaging protocol

A routine pre-TAVR protocol CTA was performed with retrospective ECG gating (10 phases throughout the cardiac cycle) and dose modulation in all cases [18]. CT examinations were performed on two 64-slice CT scanners (Discovery C750 HD, GE Healthcare, Milwaukee Wisconsin). A volume of 80-100 mL of commercially available intravenous ioversol 320 mg/mL contrast was injected at 5 mL/sec followed by 50 mL of normal saline. The CT scanner detector collimation width was 0.625 mm, detector coverage was 40 mm, and the slice thickness was 0.625 mm. Gantry rotation time was 0.35 sec and the scan pitch was 0.16-0.2 depending on heart rate. The tube voltage was fixed at 100 kVp and 120 kVp for patients with a body mass index of ≤30 and >30, respectively. Maximum tube current ranged from 400 and 700 mA with ECG-gated dose modulation reducing the tube current to 20% maximum between 80-20% of the R-R interval. Images were reconstructed using a standard algorithm at 10% intervals throughout the cardiac cycle, with a slice thickness of 0.625 mm. This protocol resulted in a mean radiation exposure of 13.0 ± 1.7 mSv. Data sets were transferred offline to stand-alone workstations for further analysis.

### CTA strain analysis

Three-dimensional strain analysis was performed as previously described, using a single segmentation of the aorta [13]. Briefly, an active-contour based segmentation of the thoracic aorta was performed from an end-diastolic CTA image series according to the approach described by Caselles et al.,[19] and implemented within a commercially available software platform ITK-SNAP [20]. No resampling of the obtained contouring was performed. The resultant 3D mesh model of the aorta was down-sampled and smoothed with a volume preserving approach,[21] using the commercially available software Meshlab [22]. Within the custom-built Matlab-based software that was previously described and validated, a velocity field was determined displacing the mesh from every n-th phase to the (n+1)-th phase for every phase from a single end-diastolic segmentation,[13] and the mean peak systolic maximum principal strain for the region of interest was computed [13]. Strain calculations were performed for each mesh element and then resolved for both the global thoracic aorta, and for each of the following segmental regions: ascending aorta (sinotubular junction to the brachiocephalic artery), aortic arch (brachiocephalic artery to the left subclavian artery) and descending thoracic aorta (left subclavian artery to the level of the diaphragm) [23]. All measurements were calculated in the form of peak principal strain amplitude (PPSA), from the Green-Lagrange strain tensor. The choice of computing strain over computing displacement allows quantification of aortic deformation without this being affected by rigid motion. This measurement describes the dominant direction of tissue lengthening, and is therefore expressed as a positive value [16].

### Echocardiographic imaging protocol

Echocardiography was performed using a standardized clinical protocol with an iE33 system (Philips Medical Systems, Andover, Maryland) and an S5-1 transducer (1–5 MHz). Measurements were made from an average of 3 cardiac cycles. Assessment of left ventricular (LV) geometry and function and aortic valve (AV) function were performed according to published guidelines [24, 25]. LV volumes and ejection fraction (EF) were measured using the biplane Simpson method, and volumes were indexed to body surface area (BSA). Peak AV pressure gradient was calculated as: (4 × [peak AV velocity^2^]), while mean pressure gradient was calculated by tracing the velocity-time integral (VTI) of the systolic transvalvular continuous-wave Doppler imaging signal. The LV outflow tact (LVOT) diameter was measured 5 mm proximal to the AV annulus. LV stroke volume was calculated according to the continuity equation as: (LVOT diameter)^2^ × 0.785 × (LVOT VTI), and indexed to BSA, while the AV area was calculated as: (LVOT diameter)^2^ × 0.785 × (LVOT VTI/AV VTI). The presence and severity of aortic valve regurgitation (AR) was graded based on multiple parameters as previously described [26]. Systemic vascular resistance was calculated as: 80 × mean arterial pressure (MAP)/cardiac output (CO), where MAP is the diastolic blood pressure + (pulse pressure/3) and CO is the LV stroke volume × heart rate [27]. Systemic arterial compliance was calculated as LV stroke volume index/pulse pressure, and systemic arterial elastance as (systolic blood pressure × 0.9)/LV stroke volume index [8]. Valvulo-arterial impedance was calculated as: (systolic blood pressure + mean aortic valve pressure gradient)/LV stroke volume index, representing the valvular and arterial factors opposing LV ejection by absorption of LV mechanical energy [8].

### Statistical analysis

Categorical variables are presented as counts with percentages, while continuous variables are expressed as the mean±standard deviation. Comparisons for continuous data were performed using 2-sample Student’s *t*-test. Multiple comparisons of CTA-based 3D aortic PPSA between aortic segments were performed using one-way ANOVA. Relationships between 3D aortic PPSA and clinical, hemodynamic and echocardiographic parameters were evaluated using univariable linear regression analysis. A multivariable linear regression model was performed to assess the independent correlates of global thoracic aorta and ascending aorta 3D PPSA; including age, gender, history of hypertension, stroke, LVEF, AV area, and valvulo-arterial impedance. The amount of variance accounted for by these correlates was derived from the global r^2^ of the model. CTA-based 3D aortic PPSA intraobserver variability was evaluated by having one experienced observer perform the analysis on 10 randomly selected patients and then blindly repeating the analysis one week later. Interobserver variability assessment was conducted by having a second experienced and blinded observer perform the analysis on the same 10 patients. Intraobserver and interobserver variability testing was evaluated by Bland Altman analysis, and coefficients of variation were calculated. Additionally, both intra- and interobserver agreement were analyzed by calculating intra-class correlation (ICC) coefficient with 95% confidence intervals (CI). All statistical analysis was performed using commercially available software (Matlab R2015b, The MathWorks, Inc., Natick, Massachusetts). A two-sided p-value of ≤0.05 was considered statistically significant.

## Results

### Study subjects

Among the 21 patients studied, all had adequate CTA image quality for 3D aortic PPSA analysis. Baseline clinical characteristics are shown in Table 1. There was a high prevalence of hypertension, with a minority of patients having prior myocardial infarction, heart failure and stroke.

**Table 1:** Baseline patient characteristics (*N*=21)

Parameter	Value
Clinical	
Age (years)	81 ± 6
Female, N (%)	6 (29%)
Height (m)	1.7 ± 0.1
Weight (kg)	80.7 ± 17.2
Body mass index (kg/m^2^)	24.8 ± 4.1
Body surface area (m^2^)	1.9 ± 0.2
Hypertension, N (%)	12 (57%)
Diabetes mellitus	3 (14%)
Hyperlipidemia	10 (48%)
Coronary artery disease, N (%)	12 (57%)
Prior myocardial infarction, N (%)	6 (29%)
Congestive heart failure, N (%)	8 (38%)
Stroke, N (%)	3 (14%)
Hemodynamic	
Heart rate (bpm)	61 ± 10
Systolic blood pressure (mmHg)	124 ± 16
Diastolic blood pressure (mmHg)	63 ± 12
Mean arterial pressure (mmHg)	87 ± 12
Pulse pressure (mmHg)	56 ± 14
Laboratory	
Hemoglobin (g/L)	129 ± 19
Creatinine (mcgmol/L)	106 ± 46
Estimated glomerular filtration rate (mL/min)	57 ± 17

Data are expressed as mean ± SD for continuous data and count (percentage) for categorical data

### Echocardiographic characteristics

Baseline echocardiographic characteristics are presented in Table 2. The mean LVEF was 52.6±14.2%. Five patients (24%) had a reduced LVEF of <45%. Four patients (19%) showed echocardiographic findings consistent with low-flow, low-gradient AS (defined as mean systolic transvalvular Doppler gradient <40 mmHg, with concomitant LV stroke volume index <35 mL/m^2^) [28]. No patient had greater than mild aortic valve regurgitation. Significantly elevated systemic vascular resistance and arterial compliance, arterial elastance and valvulo-arterial impedance were demonstrated. This is consistent with highly elevated valvular and arterial opposition to LV ejection coupled with a low stroke volume, characteristics consistent with chronic and severe AS.

**Table 2:** Baseline echocardiographic characteristics (*N*=21)

Parameter	Value
Left ventricular ejection fraction (%)	52 ± 14
Left ventricular end-diastolic volume index (ml/m^2^)	53 ± 16
Left ventricular end-systolic volume index (ml/m^2^)	26 ± 15
Left ventricular stroke volume index (ml/m^2^)	26 ± 7
Aortic valve annulus diameter (mm)	23 ± 3
Aortic valve area (cm^2^)	0.6 ± 0.3
Aortic valve mean pressure gradient (mmHg)	44 ± 11
Aortic valve peak pressure gradient (mmHg)	76 ± 18
Systemic vascular resistance (mmHg min/L)	28.9 ± 8
Systemic arterial compliance (ml/mmHg/m^2^)	1.0 ± 0.4
Systemic arterial elastance (mmHg/mL))	2.3 ± 0.7
Valvulo-arterial impedance (mmHg/mL/m^2^)	6.7 ± 2.1

Data are expressed as mean ± SD for continuous data and count (percentage) for categorical data. SD, standard deviation

### CTA 3D aortic strain

**Fig. 1:**
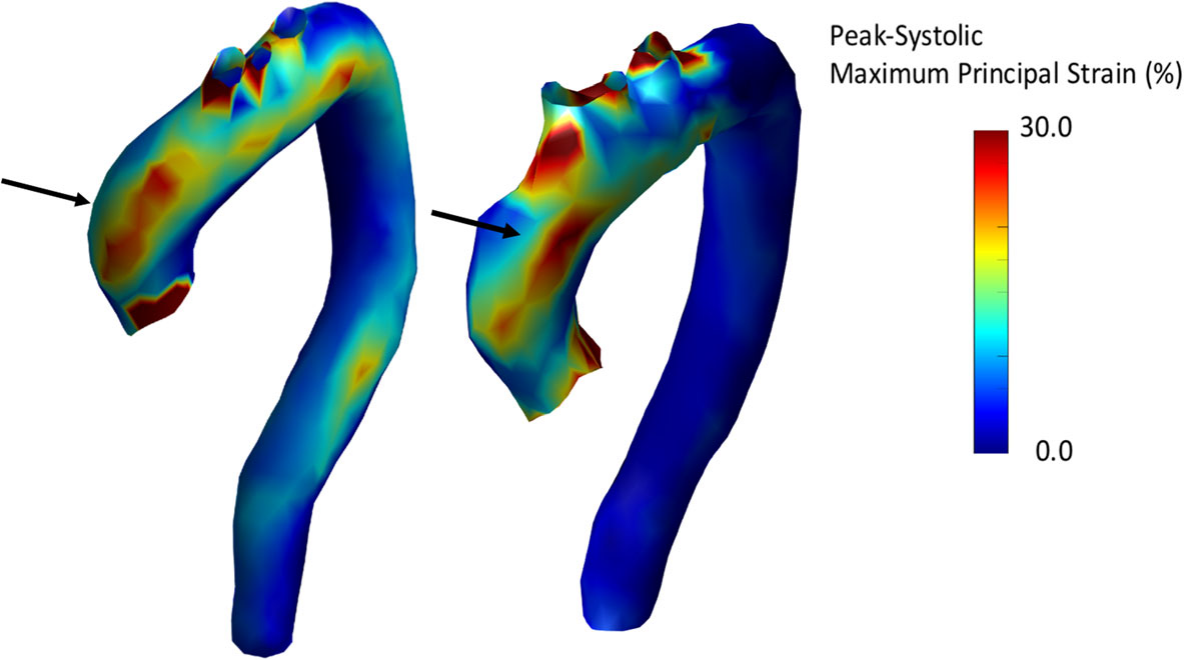
Thoracic aorta 3-dimensional peak principal strain amplitude (PPSA) calculations using ECG-gated CTA from two patients with severe aortic valve stenosis awaiting transcatheter aortic valve replacement. These images demonstrate regional heterogeneity of 3D aortic PPSA with greater amplitude strain along the ascending aorta greater curvature (arrow, consistent with AS jet directionality) and relatively reduced strain amplitude in the aortic arch and descending aorta segments. The two patients demonstrate consistent regional strain patterns

Three-dimensional aortic PPSA values for the global thoracic aorta, and for the ascending aorta, aortic arch and descending aorta segments were 6.5±3.0, 10.2±6.0, 6.1±2.9 and 3.3±1.7%, respectively, with a statistically significant difference observed between each aortic segment (p<0.0001). An example of 3D aortic PPSA from a patient with AS awaiting TAVR is provided in Fig. 1 (Additional file 1), demonstrating the typical spatial distribution of 3D PPSA observed in this population with greater strain along the greater curve of the ascending aorta segment (consistent with AS jet directionality) followed by respective reductions in strain amplitude in the aortic arch and descending aorta segments. Fig. 1 demonstrates increased strain in the following regions 1) the ascending aorta, where the aortic valve outflow jet is present, 2) in proximity of the aortic branch vessels (where either a change in curvature or concave curvature is present), and 3) in regions of relatively greater curvature, such as the aortic arch, its branches, and regions of the descending aorta.

### Correlation of 3D aortic PSSA, patient characteristics and echocardiographic measures

**Fig. 2:**
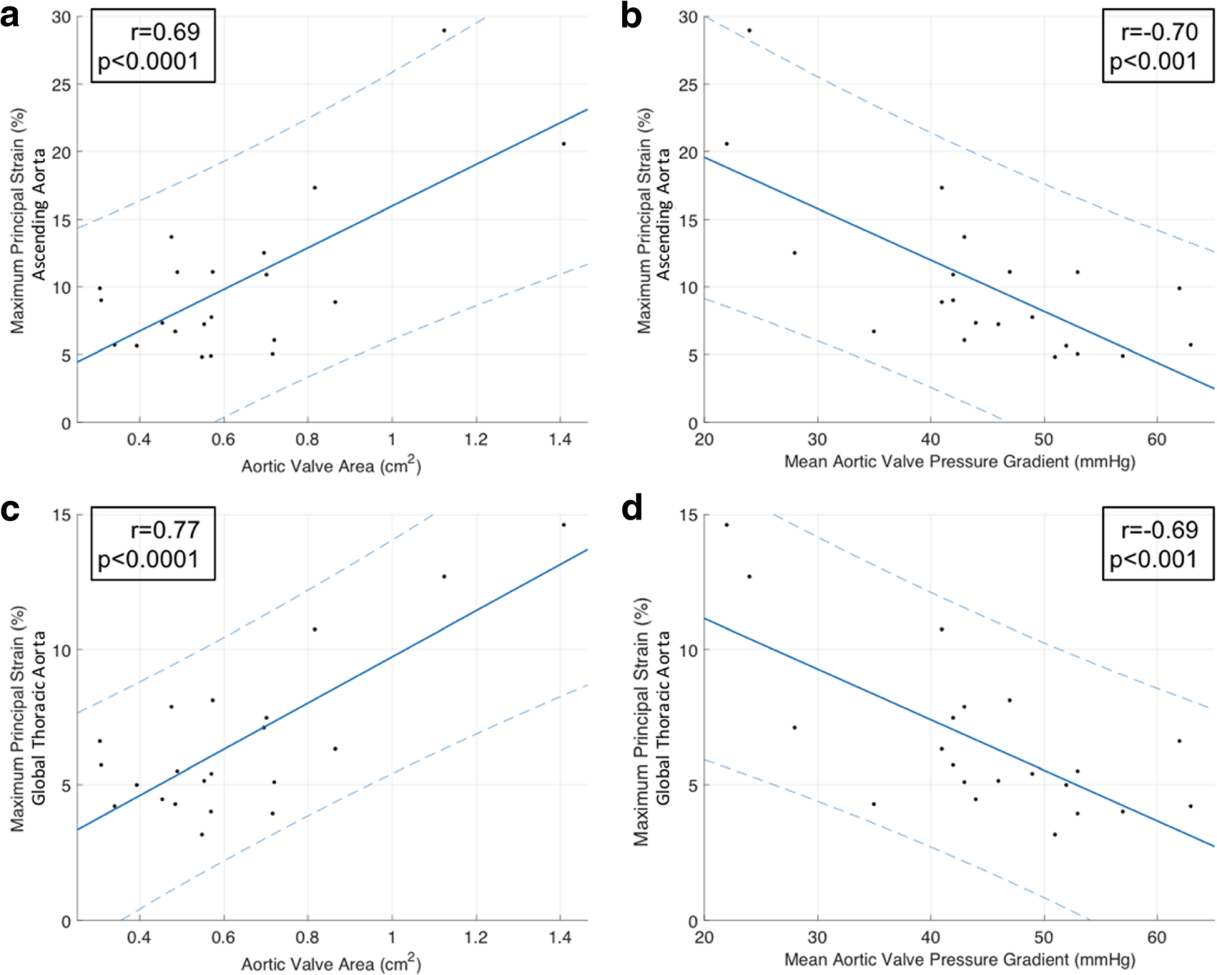
Linear regression analysis demonstrating correlation between 3-dimensional (3D) thoracic aorta peak principal strain amplitude (PPSA) using ECG-gated CTA and measures of aortic valve stenosis severity. Correlations are depicted for the ascending aorta 3D PPSA and aortic valve area (**a**) and mean aortic valve pressure gradient (**b**), and for the global thoracic aorta 3D PPSA and aortic valve area (**c**) and mean aortic valve pressure gradient (**d**). Dotted lines indicate 95% confidence intervals. *R: Pearson Correlation Rank*

As reported in Table 3, neither global thoracic aortic 3D PPSA nor ascending aorta 3D PPSA were associated with any baseline clinical or hemodynamic variables in this patient population, inclusive of LV function, with the exception of prior stroke. In contrast, significant associations were observed between 3D aortic PPSA and echocardiographic measures of AS severity (Table 3). Specifically, significant correlations were observed for both global thoracic aorta and ascending aorta 3D PPSA measurements and AV area, and mean and peak AV pressure gradients, demonstrating more impaired strain with worsening AS severity. Linear regression plots describing this relationship between ascending aorta 3D PPSA and both AV area and mean AV gradient are presented in Fig. 2a and b, respectively. Corresponding plots for global thoracic aorta 3D PPSA are presented in Fig. 2c and d, respectively.

**Table 3:** Correlations between 3-dimensional peak principal strain amplitude calculations using ECG-gated CTA of the ascending aorta and global thoracic aorta and clinical, hemodynamic and echocardiographic variables using linear regression analysis

	Ascending Aorta		Global Thoracic Aorta	
Parameter	β	*p*-value	β	*p*-value
Clinical				
Age	-0.07	0.76	0.005	0.97
Gender	2.43	0.41	0.59	0.69
Height	-0.23	0.13	-0.09	0.2650
Weight	0.04	0.61	0.02	0.68
Body mass index	0.45	0.09	0.18	0.19
Body surface area	1.04	0.86	0.57	0.84
Hypertension	1.85	0.50	-0.001	1.00
Diabetes mellitus	-2.88	0.45	-2.20	0.24
Hyperlipidemia	0.43	0.87	0.31	0.82
Coronary artery disease	-0.56	0.84	-0.08	0.95
Prior myocardial infarction	0.86	0.77	0.65	0.66
Congestive heart failure	-1.14	0.68	0.04	0.97
Stroke	9.89	0.004	4.05	0.02
Hemodynamic				
Heart rate	0.009	0.94	-0.005	0.93
Systolic blood pressure	0.03	0.71	0.02	0.68
Diastolic blood pressure	-0.09	0.41	-0.06	0.28
Mean arterial pressure	-0.05	0.69	-0.03	0.59
Pulse pressure	0.11	0.25	0.07	0.16
Echocardiographic				
LV ejection fraction	0.07	0.50	0.02	0.67
LV end-diastolic volume index	0.001	0.99	0.03	0.41
LV end-systolic volume index	-0.002	0.98	0.03	0.49
AV annulus diameter	-0.08	0.85	0.17	0.43
AV area	15.41	0.0005	8.54	0.00004
AV mean pressure gradient	-0.38	0.0004	-0.19	0.0005
AV peak pressure gradient	-0.23	0.0003	-0.11	0.0005
Systemic vascular resistance	-0.0001	0.49	-0.001	0.30
Systemic arterial compliance	-2.29	0.55	-0.97	0.61
Systemic arterial elastance	-0.64	0.73	-0.61	0.51
Valvulo-arterial impedance	0.03	0.77	0.01	0.74

AV, aortic valve; LV, left ventricle

Multivariable linear regression analyses, performed separately for global thoracic aorta and ascending aorta 3D PPSA, and clinical, hemodynamic and echocardiographic variables are reported Table 4, and again demonstrate that measures of AS severity were the most significantly correlated to reduced aortic strain. The percentage of variance of 3D aortic PPSA accounted for by the variables included in the model are reported as the global r^2^ (Table 4).

**Table 4:** Independent correlates of 3-dimensional peak principal strain amplitude calculations using ECG-gated CTA for the ascending aorta and global thoracic aorta

	Ascending Aorta		Global Thoracic Aorta	
Parameter	β	*p*-value	β	*p*-value
Age	-0.02	0.91	0.05	0.61
Gender	3.05	0.15	0.89	0.37
Hypertension	0.08	0.97	-0.65	0.51
Stroke	**8.85**	**0.03**	**3.72**	**0.046**
Heart rate	-0.04	0.75	0.003	0.95
Pulse pressure	-0.03	0.75	0.009	0.86
Left ventricular ejection fraction	-0.06	0.47	-0.04	0.31
Aortic valve area	**14.41**	**0.008**	**3.73**	**0.003**
Valvulo-arterial impedance	0.02	0.80	-0.004	0.93
Model-adjusted r^2^	0.58	0.015	0.608	0.012

The global r^2^ of the model represents the amount of variance accounted for by these correlates

### Reproducibility

**Fig. 3:**
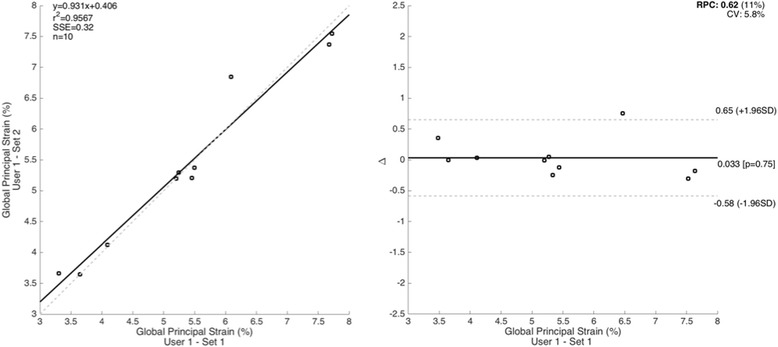
Intra-observer reproducibility of 3-dimensional thoracic aorta peak principal strain amplitude using ECG-gated CTA. The left panel depicts a regression plot while the right panel depicts a plot of differences

**Fig. 4:**
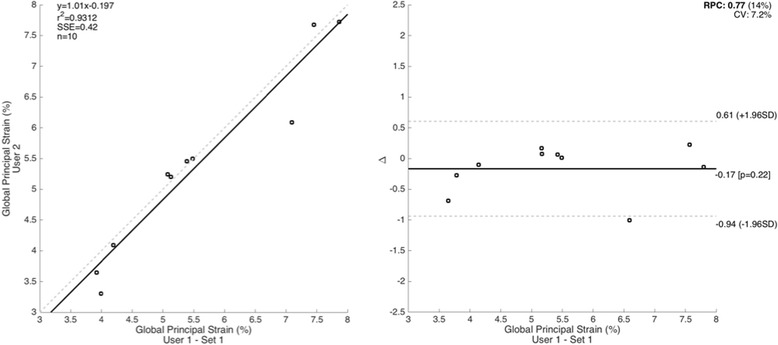
Inter-observer reproducibility of 3-dimensional thoracic aorta peak principal strain amplitude using ECG-gated CTA. The left panel depicts a regression plot while the right panel depicts a plot of differences

Bland-Altman plots documenting high intra- and inter-observer reproducibility for 3D global thoracic aorta PPSA are presented in Figs. 3 and 4, respectively. The coefficients of variation for intra- and inter-observer variability were 5.8 and 7.2%, respectively. ICC values were significant for both intra- and interobserver reproducibility (*p* <0.0001 for both). The intraobserver reproducibility value for 3D global thoracic aorta PSSA was 0.98 (0.92-0.99), while the interobserver reproducibility value was 0.96 (0.86-0.99).

## Discussion

This study demonstrates the clinical feasibility and reproducibility of 3D thoracic aorta wall principal strain mapping using routine ECG-gated CTA. Assessments of global and regional aortic biomechanics showed significantly greater strain impairment with worsening hemodynamic measures of AS severity, consistent with findings demonstrated previously by 2D aortic strain techniques [12, 29]. Accordingly, this study establishes the potential of routine ECG-gated CTA to provide reproducible measures of 3D global and regional aortic tissue health, and seeds interest for exploring such post-processing techniques across a broad range of patients with aortic disease currently evaluated by CTA.

Multiple factors are recognized to contribute to increased aortic stiffness that are well expressed in our sentinel cohort of patients with AS, including advanced age, comorbidities such as hypertension, and underlying atherosclerotic disease that affects both the AV and the arterial vascular system [9, 30]. The resultant impairment in thoracic aorta function has important biomechanical consequences on both ventricular performance and adverse remodeling, particularly among patients with AS due to impaired ventricular-arterial coupling given that both aortic stiffness and distensibility directly influence ventricular afterload incremental to that caused by valvular obstruction [3, 31]. Previous studies have demonstrated the importance of assessing biomechanical thoracic aorta properties for the prediction of adverse cardiovascular outcomes, inclusive of heart failure and all-cause and cardiovascular mortality [32–35]. Recently it has been demonstrated that vascular loading conditions may change dramatically with relief of valvular obstruction following TAVR, unmasking highly elevated aortic stiffness resulting in significant post-procedure hypertension [7]. Whether pre-procedure 3D thoracic aorta PPSA analysis incrementally improves prediction of similar adverse outcomes in this population requires further investigation. Our study found highly significant correlations between lower 3D thoracic aorta PPSA and worsening measures of AS severity. These findings suggest that the presence of severe AS has a dominant influence on aortic deformation and remodeling that may significantly outweigh concurrent physiologic factors, a relationship that has also been described in prior reports [7, 12, 36].

Localized, 2D aortic distensibility has been previously evaluated using cardiovascular magnetic resonance imaging (CMR) by measuring the difference in maximal and minimal short-axis lumen area at different phases of the cardiac cycle [32]. Despite being a simple and therefore attractive approach, this technique is unable to account for changes in longitudinal or other directions of aortic deformation, and therefore may be less sensitive for detecting clinically important alterations in thoracic aortic function [37, 38]. Bell et al. measured proximal thoracic aorta strain using CMR in N=375 subjects by constructing a longitudinal centerline that follows the aortic curvature in a 2D plane, applying intersecting perpendicular chords to characterize changes in axial diameter throughout the cardiac cycle [37]. They found that proximal aortic stiffness was over-estimated when measured using circumferential strain calculations assessed from cross-sectional imaging due to the latent effects of longitudinal strain, and that incorporation of longitudinal strain into aortic biomechanical assessment may be an important component of cardiovascular risk assessment. Our technique derives 3D PPSA and therefore is not subject to the limitations of single-plane assessment, incorporating the 3D properties of aortic deformation without the need for further correction. Principal strain analysis has been previously used to characterize the complex fibre interactions occurring during ventricular contraction,[16] and this same approach may be beneficial for providing a more physiologic metric of thoracic aorta deformation in AS and other cardiovascular diseases.

Previous reports have investigated 2D speckle-tracking echocardiography-based strain calculations of the thoracic aorta, postulating that strain is an accurate marker of aortic stiffness [39, 40]. The association demonstrated between aortic strain and collagen content provides further support for its value as a surrogate marker of vascular stiffness [41]. Teixeira et al. reported 2D circumferential ascending aorta strain values corrected for blood pressure were highly correlated with stroke volume and valvulo-arterial impedance in AS patients, concluding that both vessel wall and flow properties influence strain values [39]. Despite being widely available, echocardiography remains subject to coverage limitations and acoustic artifact for thoracic aorta imaging, particularly for multi-planar or 3D acquisitions, which may present challenges for accuracy and reproducibility.

Expanding interest in the study of ECG-gated CTA for the evaluation of vascular deformation is related to its full anatomic coverage of the thoracic aorta coupled with its superior spatial resolution, providing a dynamic isotropic dataset appropriate for quantitative biomechanical assessments [12]. Previous studies have used CTA to calculate circumferential and longitudinal 2D thoracic aorta strain based upon differences in vessel caliber throughout the cardiac cycle [10, 11, 15, 37]. More recently, Mileto et al. demonstrated the feasibility of performing aortic strain calculation at seven discrete locations using a deformable, motion-coherent modelling approach based on ECG-gated CTA acquisitions in a large cohort of patients (N=250) undergoing AV replacement [12]. Our technique offers a complimentary approach to provide a contiguous 3D principal strain analysis along the thoracic aortic. This provides a comprehensive spatial evaluation of deformation that is independent of aortic shape and orientation. We have identified regions of increased aortic wall strain in the aortic root and proximal ascending aorta that we speculate is secondary to the high velocity aortic valve outflow jet present in this population with severe AS referred for TAVR. Additionally, we have located high-strain regions in correspondence of the vessel branches, which are regions where curvature changes rapidly and can be concave. Our strain findings are consistent with previously described aortic regions of increased wall strain found in computer models [42, 43], ex-vivo optical strain measurements [44], and multi-segmentation based in-vivo strain analysis [14]. The need to quantify this inhomogeneity has been recently described [45]. Further, improved potential exists for inter-study registration given that a full volumetric evaluation is achieved, eliminating reliance on correct identification of specific anatomic landmarks [46].

### Limitations

This study was a single-center feasibility study in a small number of subjects. Therefore, this technique requires validation in broader patient cohorts with a wider range of aortic disease. Due to the exposure to ionizing radiation and iodinated contrast dye required for this CTA-based technique, a control population was not recruited for comparison with the TAVR population in this analysis. Such comparison is an important consideration for future research into the clinical utility of this approach. However, future aims will include establishing reference values from broader registry data across a range of age, sex and comorbidity. Although such analyses were beyond the scope of this analysis, our approach was previously validated against ground-truth strain data obtained from a digital phantom [13]. Additionally, this study was not longitudinal, and therefore the prognostic value of aortic 3D strain analysis is uncertain. Further research is needed to determine the correlation between 3D aortic PSSA and clinical outcomes. Finally, we were unable to perform CTA and echocardiography in the same imaging session, therefore we cannot exclude the potential for altered loading conditions that may confound comparison of results between these two modalities.

## Conclusions

Three-dimensional aortic wall principal strain analysis from routine, ECG-gated CTA is clinically feasible and shows good reproducibility. In this cohort of patients with severe AS referred for TAVR, lower 3D PPSA values were appropriately identified in patients with greater hemodynamic severity of AS, as assessed by echocardiography. Future research is warranted to explore the value of this novel technique in other patient cohorts with aortic disease routinely imaged by ECG-gated CTA.

## Additional file

Additional file 1: **Figure S1.** Mesh and Corresponding CTA view throughout the cardiac cycle, for two separate subjects. For each patient, the upper panels depict the progression of the maximum principal strain distribution throughout the cardiac cycle. The lower panes depict the evolution of the aorta as seen in a CTA plane. (PNG 891 kb)

## Abbreviations

2D: 2-dimensional
3D: 3-dimensional
ANOVA: Analysis of variance
AR: Aortic valve regurgitation
AS: Aortic stenosis
AV: Aortic valve
AVA: Aortic valve area
BMI: Body mass index
BSA: Body surface area
CO: Cardiac output
CTA: Computerized tomography angiography
ECG: Electrocardiography
LV: Left ventricle
LVEF: Left ventricular ejection fraction
LVOT: Left ventricle outflow tract
MAP: Mean aortic pressure
MG: Mean gradient
PPSA: Peak principal strain amplitude
SD: Standard deviation
TAVR: Transcatheter aortic valve replacement
VTI: Velocity time integral
