# Supplementary figures and images for: Three-dimensional thoracic aorta principal strain analysis from routine ECG-gated computerized tomography: feasibility in patients undergoing transcatheter aortic valve replacement
Source: BMC Cardiovasc Disord. 2018 May 2;18:76. doi: 10.1186/s12872-018-0818-0 (PMC5932860; doi:10.1186/s12872-018-0818-0)

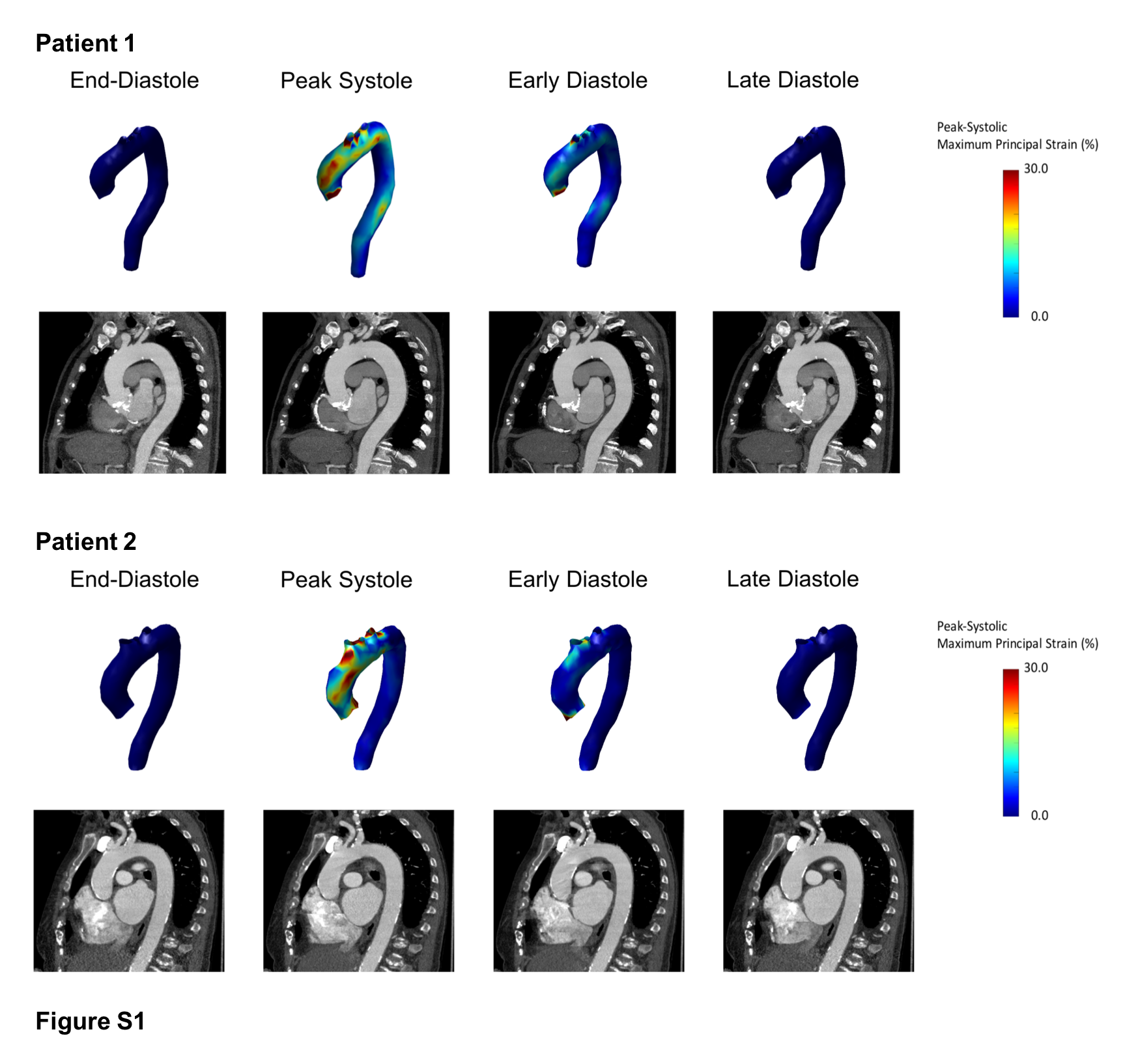

Supplement: Supplementary file 1 — Figure S1. Mesh and Corresponding CTA view throughout the cardiac cycle, for two separate subjects. For each patient, the upper panels depict the progression of the maximum principal strain distribution throughout the cardiac cycle. The lower panes depict the evolution of the aorta as seen in a CTA plane. (PNG 891 kb) [file 12872_2018_818_MOESM1_ESM.png]
